# Identification of a peptide inhibitor for the histone methyltransferase WHSC1

**DOI:** 10.1371/journal.pone.0197082

**Published:** 2018-05-09

**Authors:** Michael J. Morrison, P. Ann Boriack-Sjodin, Kerren K. Swinger, Tim J. Wigle, Dipti Sadalge, Kevin W. Kuntz, Margaret Porter Scott, William P. Janzen, Richard Chesworth, Kenneth W. Duncan, Darren M. Harvey, John W. Lampe, Lorna H. Mitchell, Robert A. Copeland

**Affiliations:** Epizyme Inc., Cambridge, Massachusetts, United States of America; Universität Stuttgart, GERMANY

## Abstract

WHSC1 is a histone methyltransferase that is responsible for mono- and dimethylation of lysine 36 on histone H3 and has been implicated as a driver in a variety of hematological and solid tumors. Currently, there is a complete lack of validated chemical matter for this important drug discovery target. Herein we report on the first fully validated WHSC1 inhibitor, PTD2, a norleucine-containing peptide derived from the histone H4 sequence. This peptide exhibits micromolar affinity towards WHSC1 in biochemical and biophysical assays. Furthermore, a crystal structure was solved with the peptide in complex with SAM and the SET domain of WHSC1L1. This inhibitor is an important first step in creating potent, selective WHSC1 tool compounds for the purposes of understanding the complex biology in relation to human disease.

## Introduction

Epigenetic mechanisms, including post-translational modifications of histones within chromatin, regulate gene transcription in normal cells [1]. It is therefore unsurprising that dysregulation of epigenetic pathways can lead to a variety of human diseases, including cancer, metabolic disease, neurological disorders, and inflammation [2]. Protein methyltransferases are enzymes that add covalent methyl groups to arginine or lysine side chains of proteins employing S-adenosyl-L-methionine (SAM) as a cofactor. The transfer of this methyl group can result in a mono-, di-, or trimethylated lysine or conversely, a mono- or dimethylated (symmetric or asymmetric) arginine. Methylation of histones has been shown to act as both a transcriptional activator and repressor making methyltransferases attractive targets for drug discovery [3].

The NSD (Nuclear receptor SET Domain containing) family of histone methyltransferases consists of three members: NSD1, WHSC1 (also known as NSD2 or MMSET), and WHSC1L1 (also known as NSD3). These three enzymes mono- and dimethylate lysine 36 on histone H3 (H3K36) [4, 5]. The NSD family members are large, multi-domain enzymes that, in addition to the catalytic SET (Su(var), E(z) and Trithorax) domain, include known epigenetic reader domains such as PHD and PWWP domains, and these reader domains likely contribute to chromatin binding [6].

Fusion proteins with the NSD family enzymes have been implicated in several cancers. NUP98-NSD1, the fusion protein resulting from the t(5;11)(q35;p15.5) translocation, has been seen in acute myeloid leukemias [7, 8]. The t(4;14)(p16;q32) translocation puts the WHSC1 gene under the control of the IgH intronic Eu (mu) enhancer resulting in elevated WHSC1 protein expression and increased dimethylation at H3K36 [9–11]. Translocations at t(8;11)(p11.2;p15) create a NUP98-WHSC1L1 fusion seen in acute myeloid leukemia [12]. Additionally, upregulation of WHSC1 has been linked to several cancers including neuroblastoma [13], gliomas [14], and several others including bladder cancer [13] and has also been linked to increased tumor aggressiveness [15]. Furthermore, a gain-of-function WHSC1 point mutation (E1099K) has also been identified in pediatric acute lymphoblastic leukemia cell lines resulting in a dependency on mutant WHSC1 activity [16, 17]. Amplification of WHSC1L1 has been seen in breast cancer where knockdown modulated the growth and survival of these cells [18].

Given the evidence linking NSD family members and oncology, there is great interest in developing potent and selective inhibitors for one or more members of the NSD family. Structure-based methods have been extremely useful in the development of several inhibitors to histone methyltransferases, such as DOT1L [19–23], EHMT1/2 (G9a/GLP) [24–30], and SMYD2/3 [31–37] and would be valuable for the NSD family as well. Autoinhibited structures of the NSD1 [38] and WHSC1 [39] SET domains have been solved along with a structure of the WHSC1L1 SET domain in an open loop conformation (PDB code = 5UPD, Structural Genomics Consortium). All of these structures have been solved in the presence of the substrate SAM, but to date no structure of an NSD protein has been solved in the presence of an enzyme inhibitor. Inhibitors based on sinefungin, a substrate analog of the cofactor SAM, have been reported for WHSC1 but structures of these compounds were reported in a closely related enzyme, SETD2, rather than in an NSD family protein [39]. Additional studies with two known inhibitors of G9a, UNC0638 [39] and BIX-01294 [40], have not shown experimental evidence of specific binding of these compounds to the active site of the NSD family members. A specific inhibitor for any member of the NSD family would be a valuable addition to the chemical library for protein methyltransferases.

In this report, the first fully validated inhibitor of WHSC1 is presented. The norleucine-containing peptide is based on the histone protein H4 sequence surrounding residue K44 (H4K44). This peptide inhibits WHSC1 in both biochemical and biophysical assays with similar potencies. Additionally, the structure of the WHSC1L1 SET domain (residues 1054–1285) complexed with PTD2 and SAM has been solved. This structure, the first NSD family member complexed with an inhibitor, reveals the importance of the autoinhibitory loop in binding the protein substrate and further reveals how crucial loop dynamics are in the catalytic cycle of the enzyme. Additionally, this result provides further documentation of the general utility of norleucine containing peptides as inhibitors of lysine methyltransferases.

## Materials and methods

### Protein production

GST-tagged WHSC1 941–1240 (#51026) and Avi-tagged WHSC1 941–1240 (#111207) (Genbank #: NM_133330 were purchased from BPS Bioscience. Purity of the purchased protein was at least 80% as measured with the Agilent Bioanalyzer.

A WHSC1L1 construct containing amino acids 1054–1285 (Genbank #: NM_001042424.2) was cloned into a vector containing an N-terminal His-TEV tag to facilitate purification. His-TEV-WHSC1L1 (1054–1285) was produced in *E*. *coli* using IPTG to induce expression. After harvest by centrifugation, the resulting cell pellets were resuspended in Buffer A (20mM Tris, 250 mM NaCl, 5% glycerol, 2mM β-ME, pH 8.0) with 1 mM PMSF added, passed through a high pressure homogenizer, and centrifuged to remove cell debris. The supernatant was added to nickel affinity resin (Qiagen) pre-equilibrated with Buffer A and incubated for 2 hours at 4°C. The solution was then packed into a column and washed with Buffer A with 20 mM imidazole until baseline was achieved. Target protein was eluted with Buffer A with 250 mM imidazole. After overnight incubation with TEV protease at 4°C, the protein was passed over nickel affinity resin pre-equilibrated with Buffer B (20mM Tris, 50mM NaCl, 5% glycerol, 2mM β-ME, pH 8.0) and washed in the same buffer; the target protein was eluted in the flow-through and wash steps. The protein was then loaded onto a SP Sepharose FF column (GE) pre-equilibrated with Buffer B and washed with Buffer B until baseline was reached. The protein was eluted using 50mM Tris, 100mM NaCl, 1mM TCEP, pH 8.5. After concentration, the target protein was loaded onto a Superdex 200 gel filtration column pre-equilibrated with 50mM Tris, 20mM NaCl, 1mM TCEP, pH 8.5. Eluted fractions were combined, concentrated and frozen for future use. The final purity of the protein was >85% by SDS-page gel and the correct mass for the cleaved protein was obtained by mass spectrometry.

### Peptide synthesis

Peptide synthesis for PTD1 and PTD2 was performed at Biopeptide with a purity > 95% by HPLC.

### Biochemical assay

The WHSC1 941–1240 biochemical assay was performed using a radiometric format involving the transfer of a ^3^[H] methyl group from SAM to an N-terminally biotinylated histone H4 peptide (RLARRGGVKRISGLI). An optimized 1× assay buffer containing 20 mM Tris-HCl (pH 8.0), 1 mM DTT, 0.005% Tween-20, and 0.01% bovine skin gelatin was utilized. The assay was performed in a 384-well V-bottom polypropylene microplate (Greiner Bio-One) at 25°C at 10 nM enzyme under balanced substrate conditions (0.15 μM ^3^[H]-SAM, 0.85 μM unlabeled SAM and 0.5 μM peptide). The reaction was quenched after 120 minutes by adding excess (1 mM final concentration) unlabeled SAM. Quenched reactions were transferred onto streptavidin coated Flashplates (PerkinElmer), incubated for 2 hours, washed 1x with 0.1% Tween 20, and read on a Topcount instrument (PerkinElmer).

The WHSC1L1 1054–1285 biochemical assay was performed as above with 40 nM enzyme under balanced conditions (0.4 μM [^3^H]–SAM, 5.6 μM unlabeled SAM, and 0.8 μM peptide).

For determining the potency of PTD1 and PTD2, 1 μL of serially diluted peptide inhibitor (in 100% DMSO) was pre-incubated with 40 μL of enzyme for 30 minutes. The reaction was initiated by adding 10 μL substrate mix containing peptide and SAM. Concentration–response curves in units of percent inhibition were plotted as a function of inhibitor concentration and fitted in GraphPad Prism to determine the IC_50_ values using a four-parameter logistic equation. The 100% inhibition control consisted of 200 μM final concentration of PTD1, whereas the 0% inhibition control consisted of 2% DMSO.

### SPR

The SPR binding assay for Avi-tagged WHSC1 941–1240 was performed at 15°C using the Biacore T200 system (GE Healthcare, Marlborough, MA). The running buffer contained 25 mM Tris-HCl pH 8.0, 150 mM NaCl, 5 mM DTT, 0.05% Tween-20, 10 μM ZnCl_2_, 50 μM SAM, and 2% DMSO. Biotinylated WHSC1 was immobilized on a streptavidin-coated SA chip up to 3700 RU (response units). The reference cell was blocked with PEG-biotin. Peptide inhibitor was injected at a flow rate of 50 μL/min with a 3-fold, 5-point dilution series ending at a 20 μM top concentration. Association and dissociation times were 240 and 2400 seconds, respectively. Double-referenced data were analyzed globally using a 1:1 binding model in BIAevaluation software.

### Crystallography

PTD2 (2 mM) and SAM (2mM) were incubated with WHSC1L1 1054–1285 (10 mg/ml) on ice prior to crystallization. Vapor diffusion methods utilizing hanging drops with a 0.5 mL reservoir were used. 1 μL protein was added to 1 μL of reservoir solution containing 0.1 M Bis-Tris pH 6.5, 20% w/v PEG5000MME; 0.2 μL 0.1 M Sarcosine and microseeds were added to facilitate crystallization. Crystals were frozen in liquid nitrogen after incubation in a solution containing 80% reservoir/20% glycerol. Data reduction and scaling were performed with XDS [41]. Structure determination was performed using the WHSC1L1 SET domain structure (PDB code = 5UPD) and visual inspection of electron density maps. Structure refinement was completed using iterative cycles of refinement and model building using REFMAC [42] and COOT [43], respectively. Data collection and refinement statistics are shown in Table 1. The WHSC1L1 1054-1285-PTD2 structure has been deposited into the Protein Data Bank (PDB code = 6CEN).

**Table 1:** Crystallographic data collection and refinement statistics for WHSC1L1 1054–1285 with PTD2 and SAM.

PDB code	6CEN
Synchrotron/beamline	APS 22-ID-D
Space group	P2_1_
Unit cell (a, b, c, α, β, γ)	42.87, 62.66, 48.60, 90.0, 107.9, 90.0
Resolution range (Å) (Highest resolution shell)	100–1.61 (1.65–1.61)
Completeness overall (%)	99.6 (99.7)
Reflections, unique	29799
Multiplicity	3.72 (3.71)
I/σ	15 (2.5)
Rmerge_overall_^**1**^	0.059 (0.629)
*R*value _overall_ (%) ^**2**^	0.179
*R*value _free_ (%)	0.215
Non hydrogen protein atoms	1794
Non hydrogen ligand atoms (SAM)	27
Non hydrogen ligand atoms (PTD2)	42
Metal atoms (Zn)	3
Solvent molecules	226
R.m.s. deviations from ideal values	
Bond lengths (Å)	0.010
Bond angles (°)	1.6
Φ, Ψ angle distribution for residues ^**3**^	
In preferred regions (%)	98.2
In allowed regions (%)	1.8
Outliers (%)	0

^**1**^ *R*_merge_ = Σ_*hkl*_ [(Σ_*i*_ |*I*_*i*_ - ‹*I*›|)/ Σ_*i*_ *I*_*i*_]

^**2**^ *R*_value_ = Σ_*hkl*_ ||*F*_obs_|—|*F*_calc_|| / Σ_*hkl*_ |*F*_obs_|

*R*_free_ is the cross-validation *R* factor computed for the test set of 5% of unique reflections

^**3**^ Ramachandran statistics as defined by PROCHECK

## Results

NSD family members are large, multi-domain containing enzymes that, in addition to the catalytic SET domain, include known epigenetic reader domains such as PHD and PWWP domains [6, 44]. A truncated version of WHSC1 containing amino acids 941–1240, which spans the entire catalytic SET domain and additional residues on both the N- and C-termini but does not include reader domains, was used in the assay. WHSC1 is known to perform mono- and di-methylation of H3K36 [4, 5]. However, an internal peptide library covering histone protein sequences was tested to identify potential WHSC1 substrates. It was determined that peptides that included H4K44 were superior substrates for WHSC1 941–1240 than the physiological H3K36 sequence. This is consistent with experiments performed with native and recombinant histone octamers which showed H4K44 to be a superior substrate for WHSC1 in the absence of DNA [4].

**Fig 1:**
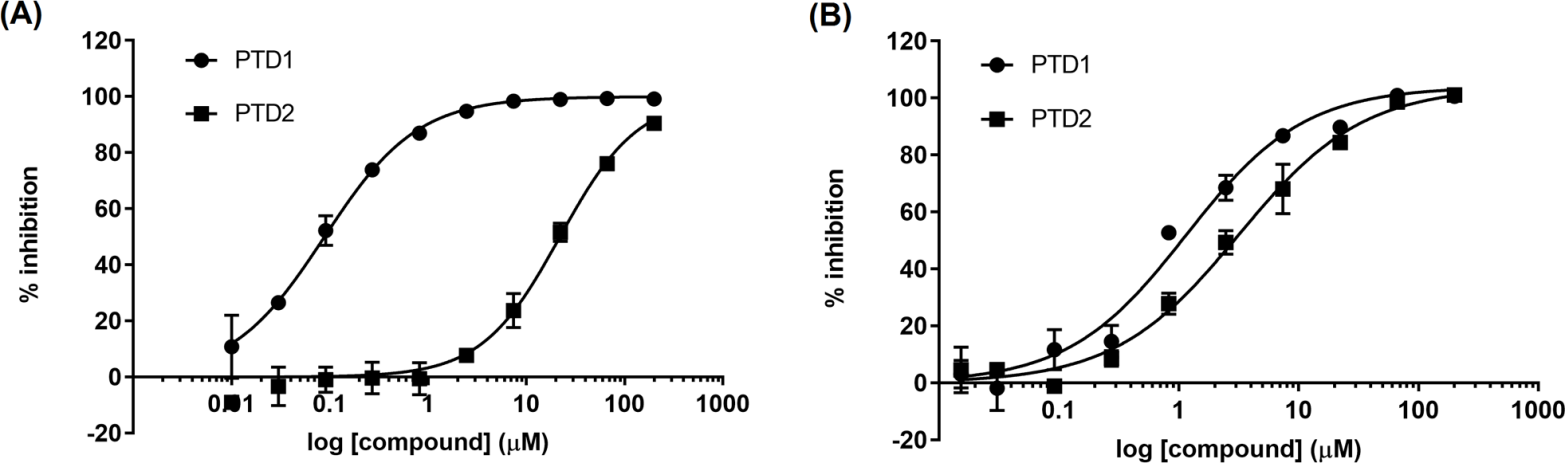
Norleucine-containing peptides can inhibit WHSC1 and WHSC1L1 activity in vitro. Representative peptide inhibitor biochemical dose-response curves for (A) WHSC1 941–1240 and (B) WHSC1L1 1054–1285. Error bars represent the standard deviation of three independent replicates. Resulting IC_50_ values are reported in Table 2.

Previous work has shown that the mutation of lysine to methionine in histone proteins can act as inhibitors for protein methyltransferases such as EZH2, SUV39H1, and G9a [45], and recent work has shown hydrophobic moieties in place of lysine can inhibit SETD2 [46, 47] and SETD8 [48]. The unnatural amino acid norleucine (Nle) can mimic the alkyl chain of lysine but is deficient in the terminal primary amine. As methylation of side chains by lysine methyltransferases requires the basic amine moiety, it was hypothesized that norleucine substituted for lysine residues would also result in a peptide that would be an inhibitor of the enzyme rather than a substrate. Two peptides were synthesized based on the H4K44 substrate sequence used in the assay, PTD1 (15 residues) and PTD2 (5 residues) (Table 2), where K44 has been replaced with Nle. These peptides were tested for inhibitory activity against a truncated construct of WHSC1 containing amino acids 941–1240 in a radiometric, biochemical assay following the transfer of ^3^H from SAM to an H4K44 peptide substrate. While the shorter 5 residue Nle peptide exhibited micromolar affinity towards WHSC1, the longer 15 residue peptide was 250-fold more potent. These data show the importance of exo site binding within the WHSC1 active site to achieve sub-micromolar potency (Fig 1 and Table 2).

**Table 2:** Biochemical and biophysical peptide inhibitor potency values for WHSC1 941–1240 and WHSC1L1 1054–1285^a^.

**Peptide**	Peptide Sequence	WHSC1 941–1240 Biochemical IC_50_ (μM)	WHSC1L1 1054–1285 Biochemical IC_50_ (μM)	WHSC1 941–1240 SPR K_D_ (μM)	WHSC1 941–1240 SPR k_on_ (M^-1^ s^-1^)	WHSC1 941–1240 SPR k_off_ (s^-1^)
PTD1	Ac-RLARRGGV[Nle]RISGLI-NH_2_	0.088 ± 0.005	1.1 ± 0.1	ND	ND	ND
PTD2	Ac-GV[Nle]RI-NH_2_	22 ± 2	3.2 ± 0.2	3.0 ± 0.3	1.7 ± 0.6 x 10^3^	5.1 ± 1.1 x 10^−3^

^a^IC_50_/K_D_ values were calculated from three independent replicates

ND = not determined

To determine the contribution of binding to WHSC1, a series of peptides were synthesized that varied the flanking position relative to the norleucine in PTD2 (Table 3). Predictably, replacement of the norleucine with alanine (PTD3) resulted in a 30-fold decrease in potency emphasizing the importance of this residue. A loss in WHSC1 potency is also observed when varying the flanking residues with respect to norleucine (Table 3) illustrating the importance of the surrounding amino acids and position of norleucine. Additionally, a series of H3K36 norleucine peptides were synthesized to determine the relative affinities with respect to the non-physiological H4K44 peptide inhibitors. As observed in Table 3, the longer 15 residue H3K36 peptide (PTD8) exhibited a substantial decrease in potency when compared with the H4K44 15 residue peptide (PTD1). This 150-fold loss in potency is extremely surprising on account of PTD8 being the physiologically relevant WHSC1 substrate sequence. The shorter 5 residue H3K36 peptide (PTD9) exhibited a more modest 6-fold decrease relative to the analogous H4K44 peptide (PTD2). This loss in potency can most likely be attributed to the replacement of isoleucine for proline resulting in a significant change in this shorter peptide sequence.

**Table 3:** Biochemical IC_50_ values for norleucine peptide inhibitors derived from the H4K44 and H3K36 sequences^a^.

Peptide	Peptide Sequence	WHSC1 941–1240 Biochemical IC_50_ (μM)	WHSC1L1 1054–1285 Biochemical IC_50_ (μM)
PTD1	Ac-RLARRGGV[Nle]RISGLI-NH_2_	0.088 ± 0.005	1.1 ± 0.1
PTD2	Ac-GV[Nle]RI-NH_2_	22 ± 2	3.2 ± 0.2
PTD3	Ac-GVARI-NH_2_	640 ± 70	> 1000
PTD4	Ac-GGV[Nle]R-NH_2_	180 ± 20	320 ± 30
PTD5	Ac-RGGV[Nle]-NH_2_	190 ± 20	110 ± 10
PTD6	Ac-V[Nle]RIS-NH_2_	110 ± 20	3.0 ± 0.3
PTD7	Ac-[Nle]RISG-NH_2_	200 ± 40	> 1000
PTD8	Ac-SAPATGGV[Nle]KPHRYR-NH_2_	13 ± 1	190 ± 30
PTD9	Ac-GV[Nle]KP-NH_2_	130 ± 20	370 ± 40

^a^IC_50_ values were calculated from three independent replicates

Inhibitor selectivity plays a crucial role in determining the molecular mechanism of action in a specific cell line. In order to understand the selectivity of this peptide series, the potency of PTD2 was determined across a panel of histone methyltransferases. While this peptide inhibitor exhibited micromolar affinity towards WHSC1, it demonstrated no enzymatic inhibition against the entire panel of methyltransferases (Table 4).

**Table 4:** PTD2 biochemical IC_50_ values for a panel of HMT enzymes.

Enzyme	Biochemical IC_50_ (μM)
WHSC1 941–1240	22 ± 2
WHSC1L1 1054–1285	3.2 ± 0.2
EZH1	>200
EZH2	>200
G9a	>200
SMYD2	>200
SETDB1	>200
PRMT6	>200

**Fig 2:**
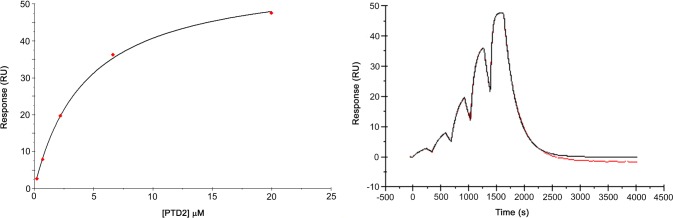
Representative sensorgram for PTD2 binding to Avi-tagged WHSC1 941–1240 from single-cycle kinetic SPR measurements. WHSC1 was immobilized on a streptavidin-coated chip and peptide inhibitor was co-injected with SAM utilizing a 3-fold, 5-point dilution series ending at a 20 μM top concentration. Data reported in Table 2 is presented as the standard deviation of three independent experiments.

**Fig 3:**
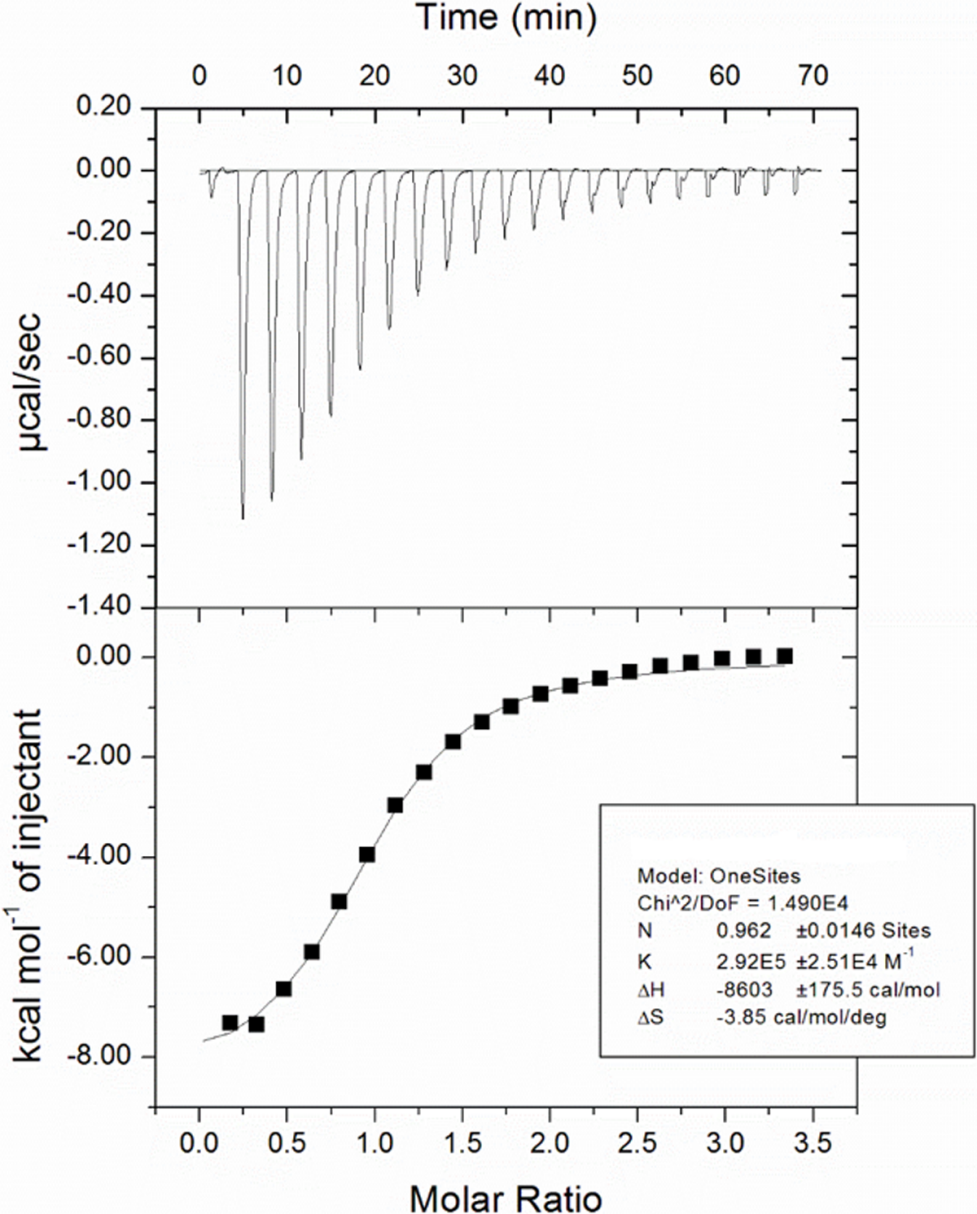
Isothermal titration calorimetry of PTD2 binding to WHSC1 941–1240. Upper panel, calorimetric trace for ligand titration; lower panel, binding isotherm from calorimetric trace. WHSC1 concentration in the cell was 10 μM supplemented with 100 μM SAM. PTD2 concentration in the syringe was 100 μM.

**Fig 4:**
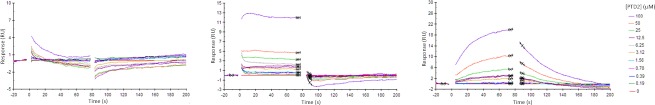
Representative SPR sensorgrams for PTD2 binding to Avi-tagged WHSC1 941–1240 in the absence or presence of SAM analogs. WHSC1 was immobilized on a streptavidin-coated chip and peptide inhibitor was either injected in the absence of cofactor (left panel), co-injected with SAH (middle panel), or co-injected with SFG (right panel) utilizing a 2-fold, 10-point dilution series ending at a 100 μM top concentration.

Biochemical enzymatic inhibition can be the result of a non-specific interaction rather than direct binding to the protein target. Therefore, an orthogonal method to measure the binding of peptides to WHSC1 was necessary. An SPR assay using an Avi-tagged version of WHSC1 941–1240 was developed and validated using cofactor SAM (S1 Fig). PTD1 exhibited non-specific matrix interactions with the SPR surface at concentrations greater than 1 μM, resulting in the inability to accurately determine peptide affinity to WHSC1 (S2 Fig). This non-specific interaction can likely be attributed to the charged state of PTD1 and its subsequent binding to the matrix on the SPR sensor chip. An increase in salt and detergent concentration was unable to mitigate this phenomenon. In contrast, sensorgrams for PTD2 showed dose dependent binding and saturation of the enzyme in single-cycle kinetics mode at affinities comparable to potency values obtained in the biochemical enzyme assay (Fig 2). To validate these results and confirm that immobilization of WHSC1 to the SPR sensor chip did not interfere with inhibitor binding, an isothermal titration calorimetry (ITC) assay was utilized. As expected, this orthogonal biophysical technique was able to corroborate the potency of PTD2 (ITC K_D_ = 3.4 μM) (Fig 3). To determine the influence of cofactor on peptide binding, PTD2 was co-injected in the presence of either S-adenosyl-L-homocysteine (SAH) or sinefungin (SFG) and in the absence of SAM (Fig 4). Importantly, the presence of SAM is essential for PTD2 potency as no binding is observed in the absence of cofactor and weak binding (K_D_ > 100 μM) in the presence of either SAH or SFG. These results suggested that the inhibition of PTD2 was the result of specific binding of the peptide in the presence of SAM to the WHSC1 protein.

**Fig 5:**
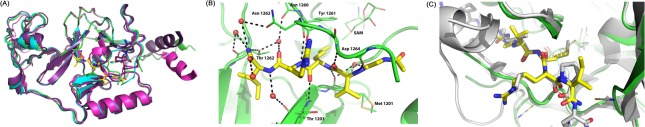
WHSC1L1 1054–1285 has a similar overall structure in relation to other NSD family proteins and can form a ternary complex with SAM and PTD2. (A) Superposition of NSD family proteins (green/yellow = WHSC1L1-PTD2 (PDB code = 6CEN); cyan = WHSC1L1 (PDB code = 5UPD); magenta = WHSC1 (PDB code = 5LSU); purple = NSD1 (PDB code = 3OOI). All protein chains are shown as ribbons; SAM and PTD2 are depicted in stick representation. (B) Structure of WHSC1L1-PTD2-SAM ternary complex. Hydrogen bonds are indicated with dashed lines. (C) Superposition of WHSC1L1-PTD2 and SETD2-H3.3 K36M (grey; PDB code = 5JJY).

In order to understand the binding mode of the peptide to the protein and to further validate these inhibitors, the structure of PTD2 was solved with a SET domain construct of WHSC1L1 comprising amino acids 1054–1285. WHSC1 and WHSC1L1 are highly homologous proteins with 45% identity (53% similarity) overall and are 78% identical (84% similar) in the sequence corresponding to the construct utilized for structure determination. Additionally, WHSC1 (PDB code = 5LSU) and WHSC1L1 (PDB code = 5UPD) [39] are extremely similar in their secondary and tertiary structures (Fig 5A) with an RMSD of 0.95 Å^2^ with comparable biochemical IC_50_ values (Table 2). Therefore, WHSC1L1 is an appropriate choice to study the binding of the PTD2.

The 1.61 Å ternary structure of WHSC1L1 1054–1285 with PTD2 and SAM confirms the inhibitor peptide binds in the histone binding pocket with the norleucine residue occupying the substrate lysine channel (Fig 5B). Significant reorganization of the autoinhibitory loop is required for peptide binding, as the peptide overlaps with the loop in both the WHSC1 and NSD1 structures, both of which are in autoinhibitory conformations. In the complexed structure, the autoinhibitory loop moves towards the C-terminus of the construct to open the binding site for the peptide. The interactions of the peptide with the protein are dominated by hydrogen bonds; with the exception of the N-terminal acetyl group and NH of the glycine residue, all backbone amide and carbonyl moieties are involved in hydrogen bonds. Met 1201 and Thr 1203 of the autoinhibitory loop, Thr 1323 and residues 1260–1264 make direct and/or water mediated interactions with peptide (Fig 5B). As previously noted, the norleucine residue points towards SAM and the terminal methyl of the unnatural amino acid is 4.1 Å from the SAM methyl group. The alkyl chain also makes hydrophobic interactions with Tyr 1262 and Phe 1259. However, the norleucine residue is extremely important for the potency of PTD2, as mutation of this residue to alanine (PTD3) ablates binding of the peptide to WHSC1 (Table 3). The binding of PTD2 to WHSC1L1 is analogous to what was seen in peptide structures with SETD2, the closest protein to the NSD family on the lysine methyltransferase phylogenic tree [49]. Despite differences in length and sequences between the histone 3.3 peptide for SETD2 (PDB code = 5JJY) and PTD2 coupled with the variances in sequence and structure between the proteins, the location of the peptide in the binding site and the conformation of the autoinhibitory loop in the WHSC1L1 structure are extremely similar in the structure of SETD2 (Fig 5C).

## Discussion

The structures currently available for the NSD family show the autoinhibitory loop can adopt multiple conformations and that these conformational changes are likely important for catalytic activity. The importance of dynamics on enzymatic activity was noted in the first report of the autoinhibited NSD1 structure and molecular dynamic simulations were performed which showed an ensemble of possible conformations [38]. More extensive work exploring loop dynamics and the effect on catalytic activity has been performed with ASH1L, another closely related methyltransferase to the NSD family [49]. Mutations to residues within the ASH1L autoinhibitory loop were previously shown to affect catalytic activity [50] and more recent experiments showed the autoinhibitory loop movements also affected the protein structure in SET-I domain, indicating the loop rearrangement may be a more complex regulation of catalytic activity [51]. Importantly, these experiments with ASH1L were performed with truncated constructs rather than with the full length proteins. Like ASH1L, the NSD proteins are large, multidomain proteins, and it is possible that regulation of the autoinhibitory loop and the conformational states available to the protein are different in the full length protein when compared to truncated constructs. Of note, the binding site for PTD2 is less than 9 Å from the C-terminus of the WHSC1L1 construct used in the crystal structure, which is not the natural C-terminus. Therefore, studies investigating the effect of construct design on catalytic activity and inhibition by peptides or small molecule compounds targeting NSD family members would be desirable.

Although the link between WHSC1 and the t(4;14)(p16;q32) translocation in multiple myeloma has been known for years, potent and selective chemical matter for WHSC1 or other NSD family members has not yet been documented. This is in contrast to other members in the protein methyltransferase superfamily, many of which have *in vitro* and/or *in vivo* tool compounds available [52] and three protein methyltransferases have compounds that have reached the clinic (EZH2, DOT1L, PRMT5; www.clinicaltrials.gov). Recently, two structurally related G9a inhibitors, UNC0638 [39] and BIX-01294 [40], have been reported to bind or inhibit one or more NSD family proteins. However, computational models of binding of BIX-01294 to the NSD co-enzyme site [40] are in direct contrast to the NMR data with UNC0638 indicating the compound interacted with the N-terminus of the SET domain construct [39]. It is possible that these two compounds differ in their interactions with WHSC1 and other NSD family members. However, a more complete understanding of these compounds and how they inhibit the enzymatic activity of NSD family members is needed before they can be considered validated chemical matter. In contrast, the work presented here shows PTD2 is the first validated inhibitor of the catalytic activity of WHSC1.

Several examples now exist of peptide inhibitors of protein methyltransferases utilizing hydrophobic moieties in place of the substrate lysine. Leucine and methionine containing peptides have been solved in SETD2 [46, 47]. Studies of inhibitory peptides for SETD8 [48] revealed a wide range of potencies could be measured by modifying the hydrophobic moiety in the lysine channel, with potencies ranging from 43 μM to ~100 nM. PTD2 is another example of this application of rational design to develop inhibitors and this approach may be useful in the design of inhibitors for other methyltransferase enzyme or other lysine binding proteins, particularly those for which small molecule drug discovery has been challenging.

Although PTD2 is a valuable asset in the toolkit for WHSC1 drug discovery efforts, additional work is needed to develop potent, selective and cell active inhibitors for WHSC1, WHSC1L1 and NSD. In SETD8, potency differences were seen by varying peptide length and the composition of residues N- and C-terminal to the core lysine channel binder [48]. The increased potency of PTD1 toward WHSC1 when compared to PTD2 may indicate that optimization of PTD2 is also possible. Although peptides typically have poor stability and cell permeability [53], advances in the field of cyclic peptides [54] and modifications such as N-methylation [55] have been successfully utilized to increase bioactivity of peptides, and this could be a potential avenue for optimization of PTD2. The structure of WHSC1L1-PTD2 may also inspire structure-based approaches for drug discovery efforts towards the NSD family, for which PTD2 will be a valuable tool for *in vitro* assay development and validation. Discovery and optimization of potent and selective inhibitors of WHSC1, WHSC1L1 and NSD1 would greatly help to understand the role of these enzymes in both normal and oncogenic cell lines and, potentially, human disease.

## Supporting information

S1 Fig Representative SPR sensorgram for SAM binding to Avi-tagged WHSC1 941–1240. (PDF)

S2 Fig Sensorgram for PTD1 showing non-specific matrix interactions with the WHSC1 941–1240 SPR surface. (PDF)
