# Supplementary figures and images for: Identification of a peptide inhibitor for the histone methyltransferase WHSC1
Source: PLoS One. 2018 May 9;13(5):e0197082. doi: 10.1371/journal.pone.0197082 (PMC5942779; doi:10.1371/journal.pone.0197082)

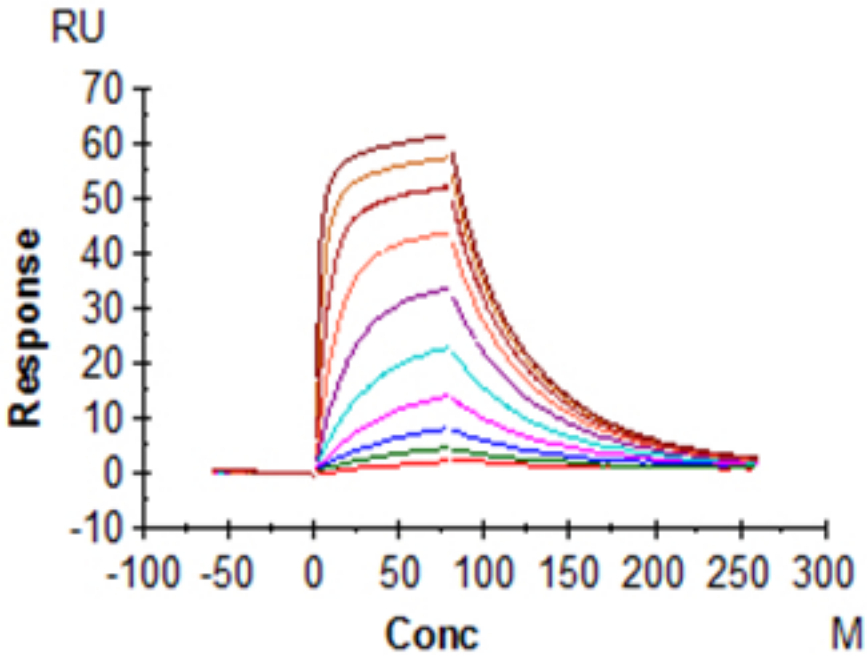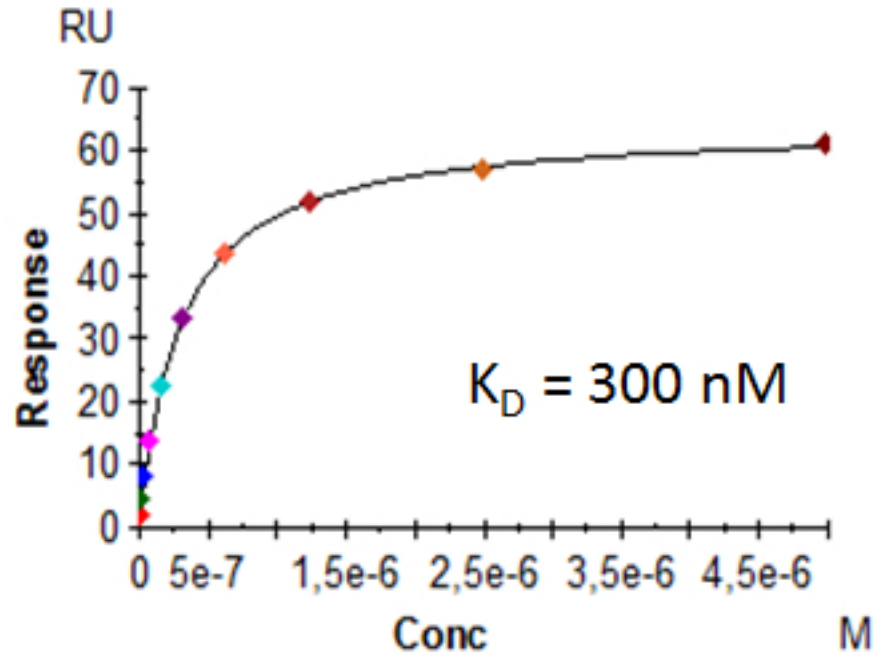

Supplement: S1 Fig — (PDF) [file pone.0197082.s001.pdf]

# WHSC1-PTD1

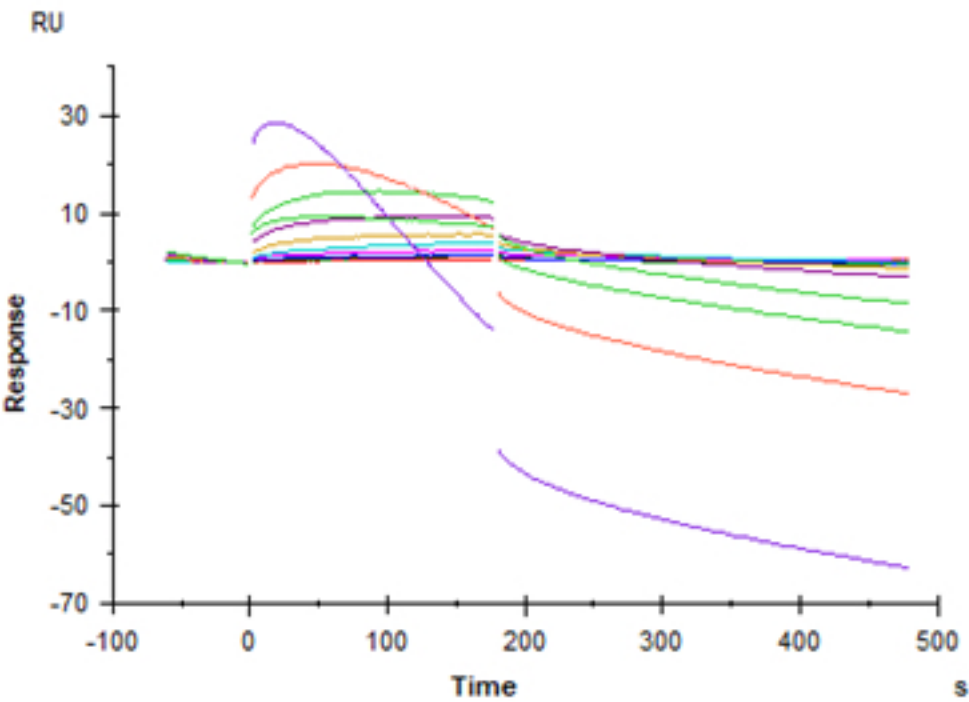

# PTD1 Reference Cell

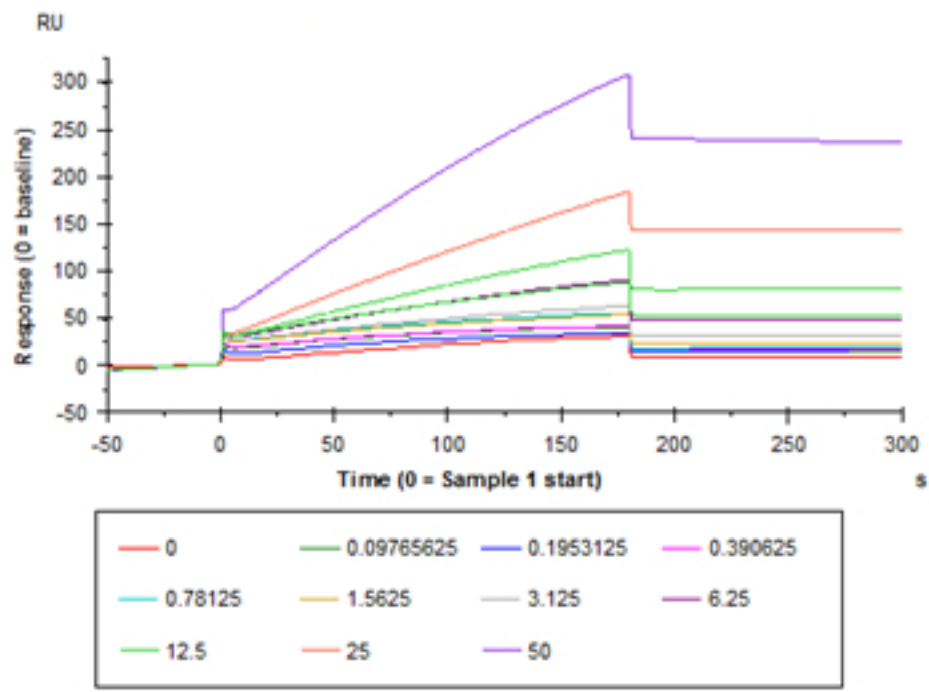

Supplement: S2 Fig — (PDF) [file pone.0197082.s002.pdf]
